# Worksite environment physical activity and healthy food choices: measurement of the worksite food and physical activity environment at four metropolitan bus garages

**DOI:** 10.1186/1479-5868-4-17

**Published:** 2007-05-11

**Authors:** Scott T Shimotsu, Simone A French, Anne F Gerlach, Peter J Hannan

**Affiliations:** 1 Division of Epidemiology & Community Health, University of Minnesota, Minnesota, USA

## Abstract

**Background:**

The present research describes a measure of the worksite environment for food, physical activity and weight management. The worksite environment measure (WEM instrument) was developed for the Route H Study, a worksite environmental intervention for weight gain prevention in four metro transit bus garages in Minneapolis-St. Paul.

**Methods:**

Two trained raters visited each of the four bus garages and independently completed the WEM. Food, physical activity and weight management-related items were observed and recorded on a structured form. Inter-rater reliability was computed at the item level using a simple percentage agreement.

**Results:**

The WEM showed high inter-rater reliability for the number and presence of food-related items. All garages had vending machines, microwaves and refrigerators. Assessment of the physical activity environment yielded similar reliability for the number and presence/absence of fitness items. Each garage had a fitness room (average of 4.3 items of fitness equipment). All garages had at least one stationary bike and treadmill. Three garages had at least one weighing scale available. There were no designated walking areas inside or outside. There were on average < 1 food stores or restaurants within sight of each garage. Few vending machine food and beverage items met criteria for healthful choices (15% of the vending machine foods; 26% of the vending machine beverages). The garage environment was perceived to be not supportive of healthy food choices, physical activity and weight management; 52% reported that it was hard to get fruits and vegetables in the garages, and 62% agreed that it was hard to be physically active in the garages.

**Conclusion:**

The WEM is a reliable measure of the worksite nutrition, physical activity, and weight management environment that can be used to assess changes in the work environment.

## Background

Overweight and obesity are increasing in the US population at an alarming rate. During the past decade, the prevalence of obesity increased by 40.6%, from 22.9% in 1988 to 32.2% in 2003–2004 [1-3]. According to recent national data, the current prevalence of overweight among US adults ages 20 years and older is 66% [3].

High economic costs are incurred as a result of the obesity epidemic. In 1995, the total economic cost attributable to obesity was $99 billion; of this, approximately $51.6 million was for direct medical costs (e.g. hospital and physician services, medications) [4]. The indirect costs of obesity included the cost of lost productivity ($3.9 billion) and days of lost work ($39.2 million) [5]. Annual US obesity-attributable medical expenditures in 2003 were estimated to be $75 billion [5].

Since employers often bear the financial burden of obesity-related costs, they often are motivated to offer worksite health behavior interventions that may prevent negative obesity-related health outcomes and thus reduce long-term healthcare costs [6,7]. Worksite interventions have the potential for broad reach since 62.8% of the US civilian labor force is employed [8,9]. Many workers spend a significant proportion of their day at the worksite, so the potential exists to be exposed to interventions in a high dose over time [10].

Environmental influences are widely recognized as important contributors to excess weight gain and the development of overweight and obesity [11]. Ecological models of change in health behavior provide a conceptual framework for targeting and measuring environmental variables [12]. Worksite physical environments provide employees with food and physical activity opportunities and exposures that influence individual food choices and physical activity behaviors [10]. The social environment at the worksite is also an important environmental influence on individual food and physical activity behaviors.

To date, worksite interventions for weight loss, physical activity and eating behavior change mostly have consisted of individual and group-based behavior change programs [10]. Although some studies have incorporated environmental elements into a multi-component intervention [13-15], few of these studies focused on environmental interventions as a primary intervention approach [10,14-16].

The recent focus on environmental approaches in interventions for health behavior change has highlighted the lack of available valid and reliable measures for the food and physical activity work environment. Although self-report measures of the perceived environment have been published [17-19], few objective measures of environmental exposures related to food and physical activity at the worksite are available [17-20]. Both the perceived and the objective environment appear to be important influences on food and physical activity behaviors [17-19].

The only worksite measure of the food and physical activity environment that we are aware of is the Checklist of Health Promotion Environments at Worksites (CHEW). The CHEW was developed to objectively measure worksite environmental influences that relate to a wide range of health-related behaviors [21]. The CHEW includes a broad range of behaviors (healthy eating, physical activity, alcohol consumption, and smoking) and has high inter-rater reliability. However, the measure's sensitivity to intervention effects is not known. In addition, associations between the CHEW objective environmental variables and individual-level perceived environmental variables are not known. Additional development of measures to assess health behavior-related worksite environments is clearly needed.

The purpose of the present research is to describe a measure of the worksite environment for food, physical activity and weight management behaviors. The measure was developed as part of a worksite environmental intervention for weight gain prevention [8]. This paper reports the development of the measure, inter-rater reliability, and descriptive information on the worksite food, physical activity and weight management environment. The measure will be used to describe the garage worksite environment, with regard to food, physical activity and weight management variables, and prospectively to assess change in the worksite environment on these variables.

## Methods

### Study overview

Data for the present research were collected as part of the baseline evaluation of a worksite environment intervention for weight gain prevention [8]. The Route H Study is an environmental intervention to prevent weight gain among metropolitan bus drivers in four garages within the major metropolitan Minneapolis-St. Paul area over a two-year period. A complete study description is available elsewhere [8]. Garages were paired on physical characteristics then randomized in pairs to intervention or control group by the toss of a coin. The multi-component intervention targeted the garage environment regarding behavioral opportunities for healthful food choices, physical activity and weight-management.

### Worksite environment measure

The worksite environment measure (WEM) was developed to evaluate the food, physical activity and weight-management environment of the Route H study worksites [see Additional File 1]. Based on the CHEW [21], the 105-item observational measure was designed to objectively assess the number, condition and presence/absence of specific items of the food, physical activity, weight-management, media, and social environment. The main areas inside and outside the general garage and maintenance areas were assessed (see Table 1).

**Table 1:** Descriptive data on food, physical activity, weight management, sedentary and social environment in four metro bus garages *, †

		**Garage**				Interrater **	**Agreement**	**Present/Absent ¶**
		**A**	**B**	**C**	**D**	**Number**	**Condition**	
**Food Environment**								
Vending Machines (total number)		10	12	10	10			
					
Refrigerated Food		2	3	2	2	1.00		
Snack Food		3(1)	2	2	2	0.75		
Cold Beverage		3	5	4	4	1.00		
Hot Beverage		2	2	2	2	1.00		
					
Water Cooler		2	0	0	0	1.00	1.00	
Microwave		3	5	4	5(4)	0.75	0.38	
Refrigerator		3	4(3)	3	5	0.75	0.50	
Restaurants (within sight; total number)		2	0	0	0			
					
Fast Food Restaurants		1	0	0	0	1.00		1.00
Other Restaurants		1	0	0	0	1.00		1.00
Grocery Stores		1	0	0	0	1.00		1.00
Convenience Stores		1(3)	1(0)	0	0	0.50		0.75
Food-related Media ¶		1	1(0)	1	1			0.75

**Physical Activity Environment**								
Fitness room (total number)		6	4	3	4			
					
Stationary Bike		1	1	1	1	1.00	0.75	
Treadmill		1	1	1	1	1.00	0.50	
Weight lifting machine		1(2)	1	0	1(2)	0.50	0.75	
Locker Room	Men	2	2	2	2			1.00
	Women	2	2	2	2			1.00
Shower Room	Men	1	1	2	1			1.00
	Women	1	1	2	1			1.00
Bicycles on Site		0	0	0	7	1.00	1.00	
Bay Walking Area		0	0	0	0	1.00	1.00	
Bay area basketball court		1	0	0	0	1.00	0.75	
Walking/bike trails (within site)		0	0	0	0			1.00
Physical activity-related media ¶		1	1	1	1			1.00

**Weight Management Environment**								
Scale		1	1	1	0	1.00	0.92	
Weight Management Media ¶		0(1)	1	1	1			0.75

**Sedentary Environment**								
Television		1	2	2	2	1.00	0.92	
Radio		3(2)	0	2(1)	2(3)	0.25	0.83	
Video game machines		1	2	1	2	1.00	0.92	
Tables ¶		1	1	1	1			1.00
Chairs ¶		1	1	1	1			1.00

**Social Environment ¶¶**								
Events where food was available		0	1	1	2			
Physical activity events and activities		0	0	0	0			
Food/Nutrition clubs, competitions, classes		0	0	0	0			
Weight Management Clubs		0	0	0	0			
Other clubs/events		0	0	0	0			

*The descriptive data are pooled across the maintenance and general areas of the worksite.

†Rater 1 is considered the gold standard. For items where there was a disagreement, the value that Rater 2 recorded is in parentheses.

**Percent Agreement = Rater 1 and Rater 2 agrees/(Agrees+Disagrees)

¶Present = 1; Absent = 0.

¶¶ Self-reported by garage coordinator. Inter-rater reliability was not measured.

Through an iterative process, items and response categories, and a standardized measurement protocol were developed and finalized based on the initial site visits to the 4 garages by the research staff. As part of the iterative developmental process, two research staff completed the WEM survey and then discussed the results with each other. Levels of detail, aggregation of items, and response categories were pilot tested and reviewed. The sites were visited several times and the process continued until a final version was agreed on by the researchers based on the level of detail needed and feasibility to obtain inter-rater reliability for observations. The final version recorded all environmental components in the garage that related to food, physical activity and weight management.

Baseline reliability data were collected independently by two trained research staff during a single site visit. The research staff who collected the reliability data were different from the developers who piloted the measure. Therefore inter-rater reliability would not be artificially improved. Each site visit required an average of 39 minutes (range: 36–40 minutes). Garage managers were contacted for permission prior to the visit. Research staff checked in with the garage managers prior to conducting the worksite assessment. The indoor garage environment had two main areas: the general driver area with a commons/break room and an exercise room; and the maintenance staff area that included a break room and separate locker rooms. The items of the WEM were grouped into sections based on the two indoor areas (general and maintenance areas) and outdoor locations. Outdoor locations measured included the immediate surrounding of a 2 block perimeter of the worksite. During site observation visits, for safety reasons, an employee escorted the research staff around the maintenance areas of the worksites. Clarification questions were asked with regard to location of fitness rooms, locker rooms, and maintenance areas.

### Food environment

The food environment was captured with 18 items. The number and condition of 10 items such as microwaves, refrigerators, and water coolers, was assessed. The format for rating the condition of items was a 4-point Likert scale (1 = good; 2 = average; 3 = poor; 4 = out of service). Examples of the food environment items included the number and type of vending machines, vending machine contents, microwaves, refrigerators, and water coolers.

### Vending machine contents

The number and type of vending machines available in the garage was recorded on the WEM measure during the site visit. The vending machine contents were recorded during a separate visit by trained research staff using a defined protocol. To describe the availability of healthful food and beverage vending choices, all of the food and beverage items were recorded and coded based on healthful criteria [22,23]. Food and beverages were coded along three nutrition dimensions: 1) low calorie (≤ 400 kcals for entrées; ≤ 150 kcals for snacks and sweets; ≤ 50 kcals for cold beverages; ≤ 120 kcals for milk); 2) low sugar (≤ 35% by weight for entrées, snacks, sweets, and cold beverages; Nuts, seeds, mints and gum were sugar-free); 3) lowfat (≤ 30% total kcals for entrées, snacks, sweets, and cold beverages). Items were coded as healthy if they met all three criteria for calories, fat and sugar (see Table 2).

**Table 2:** Vending machine items meeting criteria for fat, sugar and calories (percent)

	Garage			
Items Meeting Healthful Criteria (%)	A	B	C	D
Low Calories*	26.8	33.0	41.2	37.7
Low Sugar**	94.6	93.0	94.1	90.2
Lowfat***	67.9	82.0	72.1	75.4
Meets all 3 Healthful Criteria	21.4	27.0	36.8	32.8

*Low calorie is ≤ 400 kcals for entrées; ≤ 150 kcals for snacks and sweets; ≤ 50 kcals for cold beverages.

**Low sugar is 35% by weight for entrées, snacks, sweets, and cold beverages.

***Lowfat is ≤ 30% total kcals for entrées, snacks, sweets, and cold beverages.

### Physical activity environment

Assessment of the physical activity and sedentary-related environment included 52 items. The number and condition of radios, televisions, video game machines, and self-weighing scales was observed. The format for rating the condition of items was a 4-point Likert scale (1 = good; 2 = average; 3 = poor; 4 = out of service). The number and condition of specific exercise equipment such as stationary bikes, treadmills, and free weights was recorded. Areas such as locker rooms and showers were coded as either present or absent (1 = present, 0 = absent).

### Printed media environment

Twelve items assessed the presence or absence of nutrition, physical activity, weight-management, and other health-related printed media within and around the worksite (1 = present, 0 = absent). Media items included signage, posters, brochures, videos and bulletin boards relating to the targeted health behaviors. Other health-related media included occupational health pamphlets and mental health material. Only the outside layer of postings on the bulletin boards was measured.

### Social environment

In addition to the observed physical environment, the social environment related to food, physical activity, and weight-management behaviors was measured. The garage manager at each of the four garages was asked to answer a series of 5 questions in response to an email sent from the research staff. The managers were asked about the number of social events, clubs, or competitions held at the garage during the past three months that focused on food availability (e.g. potlucks, parties), physical activity (e.g. walking club, softball team), food and nutrition-related events (baking groups), weight-management (e.g. self-help groups, organized classes) or other social activities (e.g. cards, puzzles, knitting club). Responses were coded as yes/no (1 = yes; 0 = no) and/or the number of events (# counts). Managers were queried only once, so inter-reliability for the social environment questions was not measured.

### Outdoor physical activity and food environment

The presence of areas that may promote physical activity within the perimeter of the worksite was measured. Places in the outdoor environment were defined as those *within sight *of the worksite perimeter. "Within sight" was defined as within the view of the research observer when standing immediately outside the garage and walking around the building. Areas included designated walking areas around the immediate proximity of the worksite, parks, walking trails/sidewalks, bike trails, basketball hoops/court, and gardens. To assess the external food environment, the presence of fast food restaurants, sit-down restaurants, convenience stores and grocery stores was measured (yes/no and/or number counts). Research staff collected information on the presence of stores and restaurants without *prior *knowledge of the area. For example, a Target or Costco store may have a snack bar inside the premises, but would not be recorded as a food store, or restaurant using the outdoor environment measure.

### Perceived food and physical activity worksite environment

The perceived garage food and physical activity environment was measured with questions that were included on surveys completed by employees at each of the four garages (78% response rate). A summary is shown in Table 3. The 33-item perceived garage food and physical activity environment survey measured employee perceptions about the garage availability of healthy eating, physical activity, and weight management resources, activities and social support. Response formats for 30 items about perceptions of the food, physical activity and weight-management environment were Likert scaled (1 = strongly agree; 2 = agree; 3 = neither agree nor disagree; 4 = disagree; 5 = strongly disagree). Social support was measured using 3 questions that asked how supportive family, friends and co-workers were in one's efforts to make healthful food choices, be more physically active, and manage one's weight. Response options ranged from 1 to 5, from 1 = not at all supportive, to 5 = very supportive. The survey also measured the frequency of vending machine and fitness room use. Vending machine use during the past month was measured with one question for each type of vending machine (snack, cold/frozen food, hot/cold beverage). Response options were 1 time a month or less; 2–3 times/month; 1–2 times/week; 4 = 3–4 times/week; 5 = 5–6 times/week; 6 = 7 or more times/week. Past month fitness facilities use at the garage was queried (1 = never; 2 = 1–4 times; 3 = 5–10 times; 4 = 11 times or more).

**Table 3:** Individual-level perceived garage food and physical activity environment, social support of food choices, physical activity and weight management and use of garage vending machines and fitness room

	Garage			
Agree (%)*	A	B	C	D
At work it is:				
Easy to eat healthy	17.2	18.6	13.4	12.8
Easy to be physically active	31.9	35.8	25.4	24.7
Easy to manage weight	20.0	19.2	12.8	18.5
Hard to get fruit and vegetables	49.8	52.3	54.7	52.5
Hard to be physically active	59.3	61.2	61.9	64.8
At work, there is a lot of information on:				
Healthy eating	34.6	35.1	25.7	30.9
Physical Activity	31.9	41.4	30.7	28.0
Weight Management	26.7	31.6	25.6	21.1
Supportive (%)**				

Supportive (%)** to make healthful food choices				
Family	40.8	46.6	47.5	43.1
Friends	23.0	25.7	21.4	19.3
Co-Workers	26.7	12.1	6.9	11.1
to be more physically active				
Family	38.1	41.4	40.3	41.9
Friends	26.0	26.0	19.5	23.8
Co-Workers	11.0	9.6	7.0	19.4
to manage one's weight				
Family	34.9	38.6	40.1	24.9
Friends	19.4	31.3	15.8	18.1
Co-Workers	7.6	7.3	3.7	7.9

Vending Machine Use Frequency (past month; ≥ 3 times/week)				
Snack Food	27.5	33.5	33.3	32.9
Cold/frozen food	7.3	10.8	8.6	6.4
hot beverage	24.7	32.5	36.4	36.3
cold beverage	32.8	37.5	34.0	30.4
Fitness Room Use (past month; ≥ 1 time/week)				
	4.9	5.4	1.5	5.3

*The agree category combines "Strongly Agree" and "Agree. "

**The Supportive category combines "between Somewhat/Very Supportive" and "Very Supportive."

### Statistical analysis

All analyses were conducted using SAS 9.1 (SAS Institute, Cary, NC). Descriptive statistics and percent agreement for WEM items were computed. Percent agreement between 2 independent raters was calculated by combining the proportion of items in *exact *agreement divided by the total number of items. Agreements for the number, condition and presence/absence of items for the three environment domains (food, physical activity and weight-management) across the 4 garages was reported. Simple means and frequencies of perceived environment and individual-level survey variables were calculated.

## Results

### Worksite food, physical activity, weight-management, sedentary and social environment

Descriptive statistics and inter-rater reliability on the garage food, physical activity, weight-management, sedentary, and social environment are presented in Table 1. The number of food storage and preparation items was similar across garages. Several microwaves and refrigerators were available in each of the four garages. Percent of vending machine items that meet the criteria for fat, sugar and calories are presented in Table 2. Overall, most vending machine food and beverage items failed to meet the criteria for healthy choices due to exceeding the calorie limit.

All four garages had fitness rooms with exercise equipment available (e.g. stationary bike, treadmill). Locker rooms were present in all four garages. None of the garages had designated walking areas inside. Three garages did not have bike or walking trails or bicycles on site. Televisions and video game machines were present in all 4 garages and were located in the main commons area that had tables and chairs. Physical activity, nutrition-related, weight management and other print media were present in all four garages.

Garage managers reported garage social events within the past three months that involved food, physical activity, and weight management. In the past three months, two garages had one social event where food was available, one garage had two events where food was available, and the other garage had no events in the past three months. No other physical activity-related; food/nutrition; weight-management; or other clubs; competitions, fundraisers or classes were held at any of the garages during the past three months.

Table 1 also reports characteristics of the outdoor food and physical activity environment. Overall, few food and physical activity resources were available near the garages. None of the garages had parks, walking trails, health clubs or bike trails within sight of the garage perimeter. In general, the garages had limited food sources and options to purchase food in its locale.

### Inter-rater reliability

High inter-rater reliability was observed for the number and presence/absence categories of the food, physical activity, weight management, sedentary and social environments (Table 1). Generally, the number of items had higher inter-rater reliability than ratings of the condition of items across environment content areas. Food environment-related variables such as the number of refrigerated food, cold and hot beverage vending machines, water coolers, presence of restaurants and grocery stores around the garage perimeter, scales, bicycles, bay walking areas, and basketball courts were counted with 100% agreement between raters. Items counted with lower agreement included perimeter convenience stores, and nutrition-related and weight management-related media.

Condition of items in the food environment and physical activity environment were not as reliably assessed. Counting fitness room equipment (e.g. stationary bike, treadmill) and the presence/absence of lockers and showers were reliable (all 100%), but condition of physical activity environment items such as treadmill condition (50%), stationary bikes (75%) and weight lifting machines (75%) showed moderate reliability between raters.

### Perceived environment

Table 3 shows the frequencies for the employee perceived environment related to the garage food, physical activity and weight management environment. Overall, the garage environment was perceived to be not supportive of healthy food choices, physical activity, and weight management. On average, 52% of the employees reported that it was hard to get fruits and vegetables in the garages; 62% reported that it was hard to be physically active in the garages. Overall perceived social support for healthy food, physical activity and weight management was modest (Table 3). On average, employees reported that family members were more supportive than either friends or co-workers of healthful food choices, physical activity and weight management efforts.

## Discussion

The WEM is a reliable instrument to measure the food, physical activity, and weight-management environment in the worksite. The measure provides an easy to use instrument that can objectively capture specific aspects of the worksite environment related to food, physical activity and weight management. Such measures are useful to describe food and physical activity environmental challenges and help to identify opportunities for worksite environmental changes that will support healthful food and physical activity-related behaviors among employees. For example, few opportunities for healthful eating were available at the garages; overall, only a few vending machine foods and beverages met healthful criteria. Also, there were few restaurants or food stores within sight of the worksite. However, all worksites had refrigerators and microwaves, so employees could bring their own food. Few food-related social events were reported, but it may be possible that the measurement of the social aspects was not well-captured by the WEM. Although the WEM captured number of events, the healthful quality of events (e.g. cakes and food brought in for celebrations) could not be assessed.

Overall, the garage environment was perceived as providing low levels of social support for healthy food choices, physical activity and weight management. Perceived levels of social support were in agreement with the objective worksite measures. Perception of low fruit and vegetable availability paralleled the lack of healthful food choices in the vending machines. Each garage did have physical activity equipment, but the fitness rooms were not frequently used by the employees. Availability of refrigerators, microwaves, and fitness equipment suggest that there exist some opportunities to promote healthy food and physical activity behaviors at the garage. Social support was perceived as low to modest; perhaps the increase of social support (improving the support between co-workers) combined with greater environmental opportunities could synergistically promote healthy food and physical activity behaviors among drivers.

The WEM was adapted from the previous worksite environment measure, the CHEW [21]. The CHEW was developed to evaluate several behaviors including healthy eating, physical activity, alcohol consumption, and smoking. Although the CHEW is broad in scope, its ability to detect intervention effects is not known. The WEM was influenced by the CHEW's design, but was developed to capture a more targeted array of worksite environment dimensions, specifically food, physical activity, and weight management [8]. Although the present results are limited to a specific workplace type (bus garages), the measure could be adapted to other types of worksites. The WEM may need to be modified for worksites that have cafeterias, coffee shops, or stairways. Many worksites have vending machines and refrigerators, but may or may not have fitness rooms, food preparation areas or cafeterias. Worksite environment food, physical activity, and weight-management measures may need to be tailored for each research purpose and may need to be modified with regard to the homogeneity or diversity of specific workplaces. It may be beneficial to focus on measurement of key behavioral domains of the worksite environment most relevant to the target behaviors of interest. The WEM will be used to evaluate changes from the Route H intervention following the first year. Finally, follow-up should be conducted to evaluate whether the WEM is sensitive to secular changes and planned interventions over time.

The WEM has several strengths: the measure is reliable and objectively captures the garage food, physical activity and weight management environment. Both driver and maintenance areas as well as inside and outside the garage were reliably assessed. High inter-rater reliability of targeted environmental variables was observed, especially for counting items. In addition to objectively measuring types of items and several locations of the garage, the WEM also assessed the social environment and can be used as a process measure for obesity prevention interventions.

One limitation of the measure includes the difficulty in measuring the subjective and aesthetic qualities of the worksite. The measure did not reliably measure the condition of items (e.g. fitness room attractiveness). Another limitation is that as worksites have diverse features, settings, and types, the WEM may not be generalizable to other worksites. Also, it would be helpful to have a measure to assess factors that might increase employee motivation to use existing worksite environment resources. It may be important to evaluate the level of an item's utility and assess use for individuals (e.g. use of a refrigerator for food storage/preparation; motivation to use specific fitness equipment).

## Conclusion

The WEM is a reliable measure of the worksite nutrition, physical activity, and weight management environment that can be used to assess changes in the work environment.

## Competing interests

The authors have no competing interests to report. STS wrote the manuscript, conducted the data analysis, contributed to the study design, and incorporated input from all other authors on the manuscript. SAF developed and directed the overall study and contributed to the manuscript writing. AFG contributed to the study design, development and manuscript writing. PJH contributed to the study design, data analysis and manuscript writing.

## Supplementary Material

Additional File 1 The Worksite Environment Measure (WEM). The file provided is the worksite environment measure described in this paper. Click here for file
