# Supplementary material for: Worksite environment physical activity and healthy food choices: measurement of the worksite food and physical activity environment at four metropolitan bus garages
Source: Int J Behav Nutr Phys Act. 2007 May 11;4:17. doi: 10.1186/1479-5868-4-17 (PMC1876805; doi:10.1186/1479-5868-4-17)
Supplement: Additional File 1 — The Worksite Environment Measure (WEM). The file provided is the worksite environment measure described in this paper. [file 1479-5868-4-17-S1.pdf]

OPERATOR

Garage 1 2 3 4 5 14

Date ____ ____/____ ____/____ ____ 30

Route H ID: ☐☐ 15

Garage Environmental Assessment Form: General Garage and Operator Indoor Areas

Observer Instructions: Complete one form for the operator/general garage areas (exclude maintenance area).

1. COMMONS/BREAK ROOM

	Number of items	Condition of items*	Notes	
a. Water cooler	☐☐	☐	______	36
b. Microwave	☐☐	☐	______	39
c. Refrigerator/freezer	☐☐	☐	______	42
d. Stove	☐☐	☐	______	45
e. Sink	☐☐	☐	______	48
f. Food cart	☐☐	☐	______	51
g. Radio	☐☐	☐	______	54
h. Television	☐☐	☐	______	57
i. Video game machine	☐☐	☐	______	60
j. Scale (body weight)	☐☐	☐	______	63
k. Vending machines				
l. *Refrigerated food*	☐☐		______	66
m. *Snack food*	☐☐		______	68
n. *Cold beverage*	☐☐		______	70
o. *Hot beverage*	☐☐		______	72

(*condition codes: 1=good, 2=average, 3=poor, 4=out of service)

1. COMMONS/BREAK ROOM (continued)	Present	Not Present	
p. Food prep/counter space	1❑	2❑	74
q. Tables	1❑	2❑	75
r. Chairs	1❑	2❑	76
s. Physical activity media	1❑	2❑	77
t. Nutrition media	1❑	2❑	78
u. Weight management media	1❑	2❑	79
v. Other health-related media	1❑	2❑	80

2. EXERCISE ROOM

1❑ Not applicable, go to next section 81

	Number of items	Condition of items*	Notes	
a. Indoor bike	☐☐	☐	____	82
b. Row machine	☐☐	☐	____	85
c. Elliptical machine	☐☐	☐	____	88
d. Treadmill	☐☐	☐	____	91
e. Stair stepper	☐☐	☐	____	94
f. Weight lift machine	☐☐	☐	____	97
g. Free weights	☐☐	☐	____	100
h. Mat	☐☐	☐	____	103
i. Ball, fitness	☐☐	☐	____	106
j. Scale (body weight)	☐☐	☐	____	109
k. Radio	☐☐	☐	____	112
l. Television	☐☐	☐	____	115
m. Video game machine	☐☐	☐	____	118

(*condition codes: 1=good, 2=average, 3=poor, 4=out of service)

**2. EXERCISE ROOM (continued)**	**Present**	**Not Present**
n. Physical activity media	1❑	2❑
o. Nutrition media	1❑	2❑
p. Weight management media	1❑	2❑
q. Other health-related media	1❑	2❑

121-124

3. INSIDE GARAGE: GENERAL AREAS

	**Number of items**	**Condition of items***	**Notes**	
a. Stairwell	☐☐	☐	________________	125
b. Scale (body weight)	☐☐	☐	________________	128
c. Walking area	☐☐	☐	________________	131
d. Basketball hoop/court	☐☐	☐	________________	134
e. Bicycle	☐☐	☐	________________	137
f. Bike rack	☐☐	☐	________________	140

(*condition codes: 1=good, 2=average, 3=poor, 4=out of service)

	**Present**	**Not Present**
g. Men's lockers, operators	1❑	2❑
h. Women's lockers, operators	1❑	2❑
i. Men's showers, operators	1❑	2❑
j. Women's showers, operators	1❑	2❑

143-146

Observer: Ask Garage Coordinator for the following social environment information via email. Email the text and fill out this form after the coordinator replies to your email.

4. SOCIAL ENVIRONMENT

Garage Coordinator interviewed: ____

Event	Yes	No	Number of events (last 3 months)	Notes	
a. Parties, potlucks, work meetings, seminars, special occasions where **food** was available	1❑	2❑	☐☐		147-149
b. PA clubs, competitions, fundraisers (e.g., exercise club; fun run; 10,000 steps)	1❑	2❑	☐☐		150-152
c. Food/nutrition clubs, competitions, fundraisers, classes (e.g., recipe, gardening or cooking club)	1❑	2❑	☐☐		153-155
d. Weight management clubs, competitions, fundraisers, classes (e.g., Weight Watchers, the Big Loser)	1❑	2❑	☐☐		156-158
e. Other (please specify): ______________________________	1❑	2❑	☐☐		159-161

MAINTENANCE

Garage 1 2 3 4 5 14

Date ____ ____/____ ____/____ ____ 30

Route H ID: ☐☐ 15

Garage Environmental Assessment Form: Maintenance Area

Observer Instructions: Complete one form for the maintenance area.

1. BREAK ROOM

	Number of items	Condition of items*	Notes	
a. Water cooler	☐☐	☐	______________	36
b. Microwave	☐☐	☐	______________	39
c. Refrigerator/freezer	☐☐	☐	______________	42
d. Stove	☐☐	☐	______________	45
e. Sink	☐☐	☐	______________	48
f. Food cart	☐☐	☐	______________	51
g. Radio	☐☐	☐	______________	54
h. Television	☐☐	☐	______________	57
i. Video game machine	☐☐	☐	______________	60
j. Scale (body weight)	☐☐	☐	______________	63
k. Vending machines				
l. *Refrigerated food*	☐☐		______________	66
m. *Snack food*	☐☐		______________	68
n. *Cold beverage*	☐☐		______________	70
o. *Hot beverage*	☐☐		______________	72

(*condition codes: 1=good, 2=average, 3=poor, 4=out of service)

BREAK ROOM (continued)	Present	Not Present
p. Food prep/counter space	1❑	2❑
q. Tables	1❑	2❑
r. Chairs	1❑	2❑
s. Physical activity media	1❑	2❑
t. Nutrition media	1❑	2❑
u. Weight management media	1❑	2❑
v. Other health-related media	1❑	2❑

74-80

2. INSIDE GARAGE & BAY AREA - MAINTENANCE

	Present	Not Present
a. Men's lockers, maintenance	1❑	2❑
b. Women's lockers, maintenance	1❑	2❑
c. Men's showers, maintenance	1❑	2❑
d. Women's showers, maintenance	1❑	2❑
e. Food truck	1❑	2❑

81-85

Garage **1 2 3 4 5** 14

Date of Visit ____ ____ / ____ ____ / ____ ____ 30

Observer: [ ][ ] 15

Garage Environmental Assessment Form: Outdoor Areas

Instructions: Complete one form per garage. Include only those items that are IN SIGHT of the garage.

OUTDOOR PHYSICAL ACTIVITY ENVIRONMENT

1.	Bike racks	1❑ Yes<br>2❑ No	Notes:
2.	Walking area: garage perimeter	1❑ Yes<br>2❑ No	Notes:
3.	Park	1❑ Yes<br>2❑ No	Notes:
4.	Walking trails	1❑ Yes<br>2❑ No	Notes:
5.	Bike trails	1❑ Yes<br>2❑ No	Notes:
6.	Basketball hoop/court	1❑ Yes<br>2❑ No	Notes:
7.	Garden area	1❑ Yes<br>2❑ No	Notes:
8.	Skyways within sight	1❑ Yes<br>2❑ No	Notes:
9.	Health clubs within sight	1❑ Yes<br>2❑ No	Notes:

36-44

continues on next page

		Number	Notes	
10. Fast food restaurants	1❑ Yes<br>2❑ No	☐☐		45
11. Other restaurants	1❑ Yes<br>2❑ No	☐☐		48
12. Convenience stores	1❑ Yes<br>2❑ No	☐☐		51
13. Grocery stores	1❑ Yes<br>2❑ No	☐☐		54
